# 3D printed cylindrical capsules as a Chlorella pyrenoidosa immobilization device for removal of lead ions contamination

**DOI:** 10.3389/fchem.2022.987619

**Published:** 2022-11-30

**Authors:** Shuzhen Lan, Xinshu Xia, Zhen Liu, Yujin Yang, Qingrong Qian, Yongjin Luo, Qinghua Chen, Changlin Cao, Liren Xiao

**Affiliations:** ^1^ Engineering Research Center of Polymer Green Recycling of Ministry of Education, Fujian Normal University, Fuzhou, China; ^2^ College of Chemistry and Materials, Fujian Normal University, Fuzhou, China; ^3^ College of Environment and Resources, Fujian Normal University, Fuzhou, China

**Keywords:** 3D printed cylindrical capsules, self-floating, adsorption, immobilization, cross-linked PVA hydrogels

## Abstract

Immobilization is considered as a promising strategy toward the practical applications of powdered adsorbent. Herein, three dimensional (3D) printing cylindrical capsules with cross-linked PVA hydrogels membrane in encapsulate Chlorella pyrenoidosa (Cp) were utilized for removal of lead ions. The chemical compositions, hydrogels performance and morphologies of the membranes were determined by Fourier transformed infrared spectroscopy (FTIR), cross-linking degree, swelling degree, membrane flux and scanning electron microscopy (SEM). It is found that PVA cross-linking structure is successfully synthesized on the surface of capsule body and cap due to the presence of PVA in the filament. The lead ions adsorption capacity related to initial concentration of 50 mg/L in 48 h is reached 75.61%, revealing a good removal ability. The self-floating 3D printed capsules device also shows an excellent recovering property. After 7 runs of adsorption experiment, the lead ions adsorption ratio remains 78.56%, which will bring a broad prospect in wastewater treatment, chemical slow release along with sample preparation and separation.

## Introduction

Adsorption is considered to be one of the most effective and economical wastewater treatment methods (Singh, et al., 2018). However, adsorption can only transfer pollutants but not eliminate them completely, which is easy to cause secondary pollution (Dai, et al., 2021; Crini, et al., 2016). In particular, most of the currently developed adsorbents are micro-nano materials, which are difficult to separate from water, limiting their practical application (Tesh and Scott, 2014; Husein et al., 2021). Therefore, it is of great significance to separate and enrich pollutants economically and effectively in the process of wastewater treatment (Yao, et al., 2014).

At present, the research idea is basically to change the micro-nano adsorbent into a large volume adsorbent, which is called immobilization method. For example, the adsorbent is loaded on the framework by coating, deposition, grafting, *in-situ* synthesis and other ways (Ahmed and Jhung, 2014; Jethave, et al., 2018; Moreno-López, et al., 2019; Tahmasebi, et al., 2019; Wang, et al., 2019; Zhou, et al., 2019; Wang, et al., 2021). It is also a common immobilization method to make the adsorbent into a monolith using a binder (Salem and Akbari Sene, 2011; Novais, et al., 2016; Xiang, et al., 2017), which is similar to the method of melt blending adsorbent/polymer (Dlamini, et al., 2011; Makhetha, et al., 2016; Moja, et al., 2020). These methods are simple to prepare and easy to mass-produce, but the adsorbent is easily wrapped inside, which results in a decrease in adsorption performance. In addition, other immobilization methods such as cross-linking (Han, et al., 2014; Yu, et al., 2014; Ding, et al., 2018) and gel encapsulation (Ngomsik, et al., 2006; Tavakoli, et al., 2013) are also reported in the literature. In short, the intersection of technologies in different fields brings more options for immobilization methods.

The advantages of 3D printing technology in manufacturing complex structural materials also bring new technical inspiration for the immobilization of adsorbents. For example, DIW (Direct Ink Writing or Robocasting) provides a promising method for the preparation of adsorbent monoliths (Thakkar, et al., 2016; Couck, et al., 2017; Thakkar, et al., 2017). SLS (selective laser sintering) is employed to fabricate 3D MOF-polymer structure practical applications (Lahtinen, et al., 2019; Li, et al., 2019). FDM (Fused Deposition Modeling) is the most widely used 3D printing technology, which is used as the framework for adsorbent loading (Wang, et al., 2014; Shi, et al., 2017). More applications of FDM are the manufacture of adsorbent/polymer 3D printing filaments into adsorption devices (Bible, et al., 2018; Channell, et al., 2018; Evans, et al., 2018). With the design ability of the 3D printed structure, the adsorbent can be exposed as much as possible, which makes up for some shortcomings of the original immobilization method. But these are just improvements to existing fixation methods in reality.

Previous research in biosorption suggested that microalgae is an ideal biomaterial for the treatment of effluents with heavy metal since microalgae can effectively remove metal ions and metal complexes from solution (Ahluwalia and Goyal, 2007; Romera, et al., 2007). Chlorella pyrenoidosa (Cp) is one of the most common microalgae, and its hydroxyl and phosphoryl functional groups have a good adsorption effect on Pb(II) (Li, et al., 2017). The average particle size of Cp is mainly concentrated in the range of 10–20 μm, and the smallest particle size is close to 1 μm, which is difficult to recycle when dispersed in water.

**FIGURE 1:**
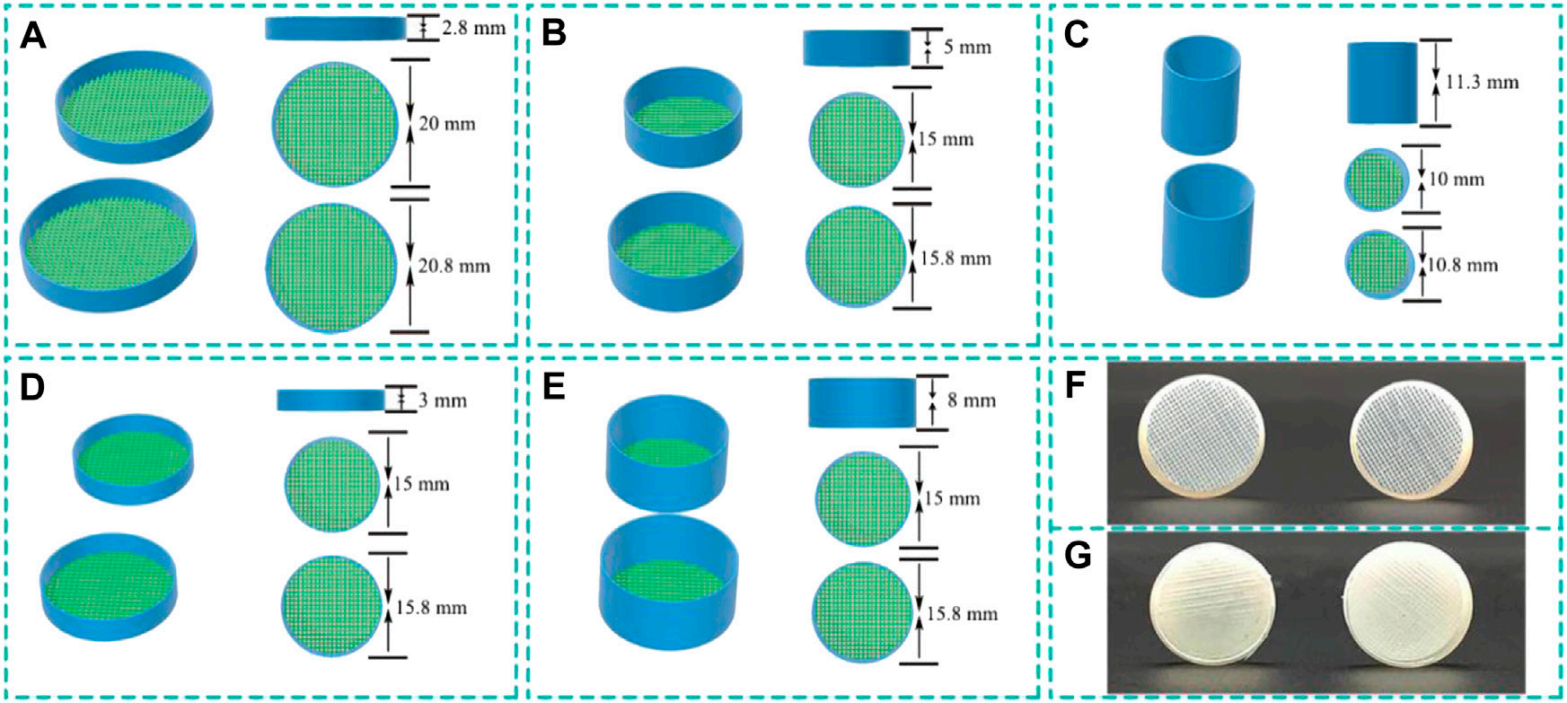
Schematic illustration and real image of the cylindrical capsule models structures. **(A-E)** Schematic illustration of h2.8d20, h3.0d15, h5.0d15, h8.0d15 and h11.3d10 with vary parameters, and **(F,G)** photographs of h5.0d15 capsule.

**FIGURE 2:**
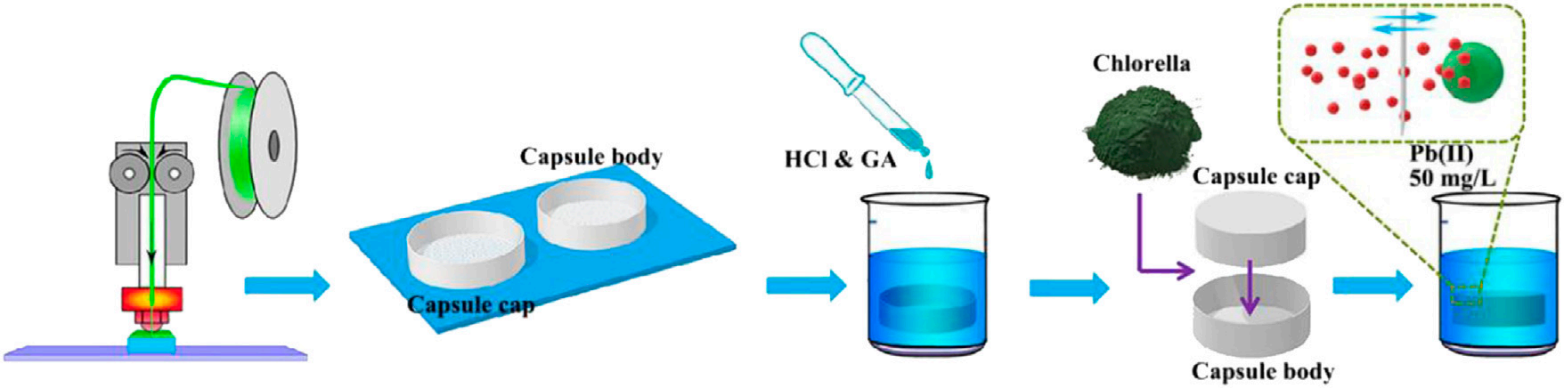
Schematic illustration of the fabrication process of cross-linked PVA cylindrical capsules structures and immobilization Cp adsorption experiment.

In this context, 3D printed cross-linked PVA cylindrical capsules, Cp and lead ions were selected as experimental objects to study the adsorption performance. Inspired by tea bags that tea powders are encapsulated in bags, we creatively proposed an alternative immobilization method, which design cylindrical capsules to encapsulate adsorbent powder by 3D printing technology with cross-linked PVA hydrogels membrane, as depicted in Figures 1, 2. The cross-linked PVA cylindrical capsules developed with self-floating can achieve stable floating on the water surface without external force, which is very beneficial for recycling. We mainly studied the effect of cross-linked PVA hydrogels membrane and cylindrical capsule structure on the adsorption performance were systematically investigated. The purpose of this study is to develop an auxiliary tool for separating and recovering powder materials.

## Materials and methods

### Materials

LAY-FOMM 60 (1.75 mm filament) was purchased from CC-Products, Köln, Germany. Chlorella pyrenoidosa (Cp) was provided from Wudi Lvqi Bioengineering Co., Ltd. Lead nitrate (analytical grade) were obtained from Sinopharm Chemical Reagent Co., Ltd. Glutaraldehyde (50 wt%) were purchased from Tianjin Fuchen Chemical Reagent Co., Ltd. Hydrochloric acid (36–38 wt%) were obtained from Shanghai Chemical Reagent General Factory.

### Preparation of capsules

The cylindrical capsule models of different structure sizes was designed by Dimensions (a 3D CAD program) and the software Cura used to slice and set various parameters. The capsule was divided into two parts (capsule body and capsule cap) that can be combined together. The capsule body was designed with vary, height (2.8, 3.0, 5.0, 8.0, and 11.3 mm) and diameter (20, 15, 15, 15, and 10 mm) parameters. The corresponding capsule cap was designed with height (2.8, 3.0, 5.0, 8.0, and 11.3 mm) and diameter (20.8, 15.8, 15.8, 15.8, and 10.8 mm), respectively. These five capsules were noted as hxdy, where x, y indicate the value of height and diameter of capsule body. The circular surfaces of the capsule body and capsule cap were designed as grid-shaped, and the remaining was fabricated as thin-walled. The schematic illustration and real image of the cylindrical capsule models structures are shown in Figure 1.

The cylindrical capsule models were fabricated on a FDM printer (M2030, Shenzhen Soongon Technology Co., Ltd., China) using the LAY-FOMM 60 filaments, which is made from a rubber-elastomeric polymer and a PVA-component. During the printing process, the nozzle temperature was set at 200°C, and the platform was kept at 60°C. The printing speed was set to 80 mm/s with a layer thickness of 0.2 mm, wall thickness of 0.4 mm, 100% in fill, and nozzle diameter of 0.20 mm.

The fabrication of cross-linked PVA Chlorella pyrenoidosa capsules structures and adsorption experiment was schematically described in Figure 2. Firstly, the grid capsule was immersed in 5 ml deionized water with 50 μL HCl and different amount of glutaraldehyde (GA) solutions (10 μL, 20 μL, 30 μL) at 25°C for 24 h, then the product dried at a gradient temperature of 30–60°C for 12 h. After the reaction, the product was placed in a drying dish for later use. The cross-linked PVA capsules were named as hxdy-z, where z indicate that the amount of GA, for example, h50d150-10. Finally the Cp was encapsulated in the capsules, labeled as hxdy-z-Cp.

### Performance testing

The cross-linking degree, swelling degree, and membrane flux of the cross-linked PVA porous membrane are obtained by Eq. 1 to Eq. 3 (Farid, et al., 2016), respectively.

$$\text{Nominal}\;\text{Cross}-\text{lingking}\;\text{degree}=\frac{\text{weight}\;\text{of}\;\text{GA}}{\text{weight}\;\text{of}\;\text{cross}-\text{linked}\;\text{PVA}}\%$$ (1)

The cross-linked PVA was pre-weighed and immersed in water at a 1:100 weight ratio. The experiments were carried out at 25°C. The sample was weighed until the swollen weight reached equilibrium.

$$\text{Swelling}\;\text{degree}=\frac{m_{\text{fp}}-m_{\text{ip}}}{m_{\text{ip}}}$$ (2)

Where m_fp_ and m_ip_ are final and initial mass of the capsule, respectively.

$$F=\frac{V}{A\Delta t}$$ (3)

Where V represents the volume of water passing through the membrane. A is the cross-sectional area of the membrane, and Δt is the filtration time.

### Characterization

The cross-linked PVA porous membrane was analyzed by Scanning Electron Microscope (Regulus 8100, Hitachi, Japan), at an operation voltage of 10 keV. The IR spectra was recorded from 4000 to 600 cm^−1^ by a Fourier transformed infrared spectroscopy (FTIR, Thermo Scientific Nicolet 5700) under room temperature (25°C).

### Adsorption experiment

Cp (0.1 g) was encapsulated in different cylindrical capsules before put into 50 ml of Pb (II) solution (50 mg/L) and rotated with a speed of 200 rpm at 25°C. The adsorption time was from 0 to 48 h. Samples were taken in different bottles according to different time intervals (2, 6, 12, 22, 32, and 48 h). As comparison, the adsorption experiment of Cp powder without encapsulation was carried out under the same experimental conditions. The Pb (II) content was directly determined by Atomic absorption spectrometer (PinA Acle 900F, Thermo Scientific, American) after sampling. All experiments were performed in triplicate. The cylindrical capsule removal efficiency to Pb (II) were calculated in Eq. 4.

$$Adsorption\;efficiency=\frac{C_{o}-C_{e}}{C_{o}}\times 100\%$$ (4)

Where C_0_ (mg·L^−1^) is the initial concentration of contaminant solution, C_e_ (mg·L^−1^) is the concentration of contaminant reach the adsorption equilibrium.

## Results and discussion

### FTIR analysis of cross-linked PVA

It is well known that the aldehyde group can cross-link reaction with the hydroxyl group in the PVA to form cross-linked PVA three-dimensional network structure (Supplementary Figure S1). The GA is a more effective cross-linking agent than other aldehydes (Wang, et al., 2005). The cross-linking reactions between PVA and GA can be intramolecular and/or intermolecular cross-links and may contain unreacted aldehyde ends (Zhang, et al., 2009). In this research, the 3D printed LAY-FOMM 60 filament containing PVA was used to fabricate cylindrical capsule. Then the PVA of the cylindrical capsule *in situ* cross-linked by GA forms cross-linked PVA porous membrane. The tortuous surface of the grid capsule can be securely anchored to the embedded PVA hydrogel to provide excellent structural integrity.

**FIGURE 3:**
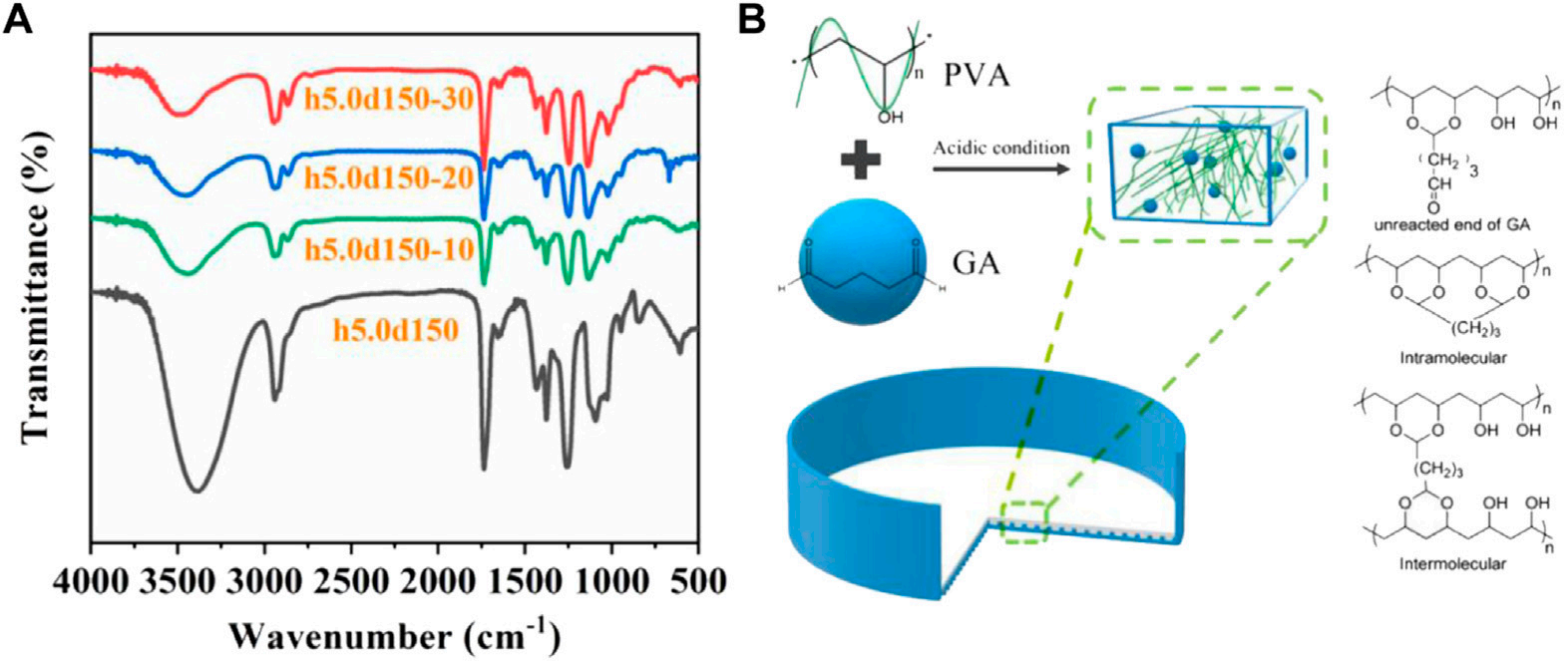
**(A)** FTIR spectra of h5.0d15, h5.0d15-10, h5.0d15-20 and h5.0d15-30; **(B)** Mechanism diagram of the acetalized cross-linking reaction between hydroxyl group of PVA and GA.

Figure 3A shows the FTIR spectra of h5.0d15, h5.0d15–10, h5.0d15–20 and h5.0d15-30. Obviously, the typical spectrum of PVA can be observed in un-crosslinked h50d150. A broad band appeared at around 3,010–3,700 cm^−1^ corresponds to the -OH stretching. The peaks at about 2,940 cm^−1^ and 1,650 cm^−1^refer to the -CH_2_- the asymmetric and symmetric stretching and the bending vibration of -O-H, respectively. After cross-linking, the -O-H adsorption peak of h5.0d15-10, h5.0d15-20, h5.0d15-30 were concentrated at 3,445 cm^−1^, 3460cm^−1^ and 3,480 cm^−1^, respectively, indicating an obvious blue shift relative to h5.0d15. This indicating that hydrogen bonding of hydroxyl groups on PVA chains was weakened after being chemically cross-linked by GA. Importantly, the adsorption bands attributed to -CH_2_- stretching mode were split into 2,940 and 2,860 cm^−1^ and the intensities of these bands were remarkably enhanced, which is due to the introduction of -O-CH_2_-O- by acetalization (Zhang, et al., 2020). In addition, the band attributed to secondary -O-H in-plane bending at 1,430 cm^−1^ was diminished as the increase of the amount of cross-linking agent. It was also found that the band attributed to C-O-C stretching at 1,020 cm^−1^ was remarkably enhanced with the increased of GA content, indicating that C-O-H transformed to C-O-C and cross-linking structure can be formed (Wang and Wang, 2016). The above FTIR analysis confirmed the acetalized reaction between hydroxyl group of PVA and GA by forming acetal bridges, as shown in Figure 3B.

### SEM analysis of cross-linked PVA

**FIGURE 4:**
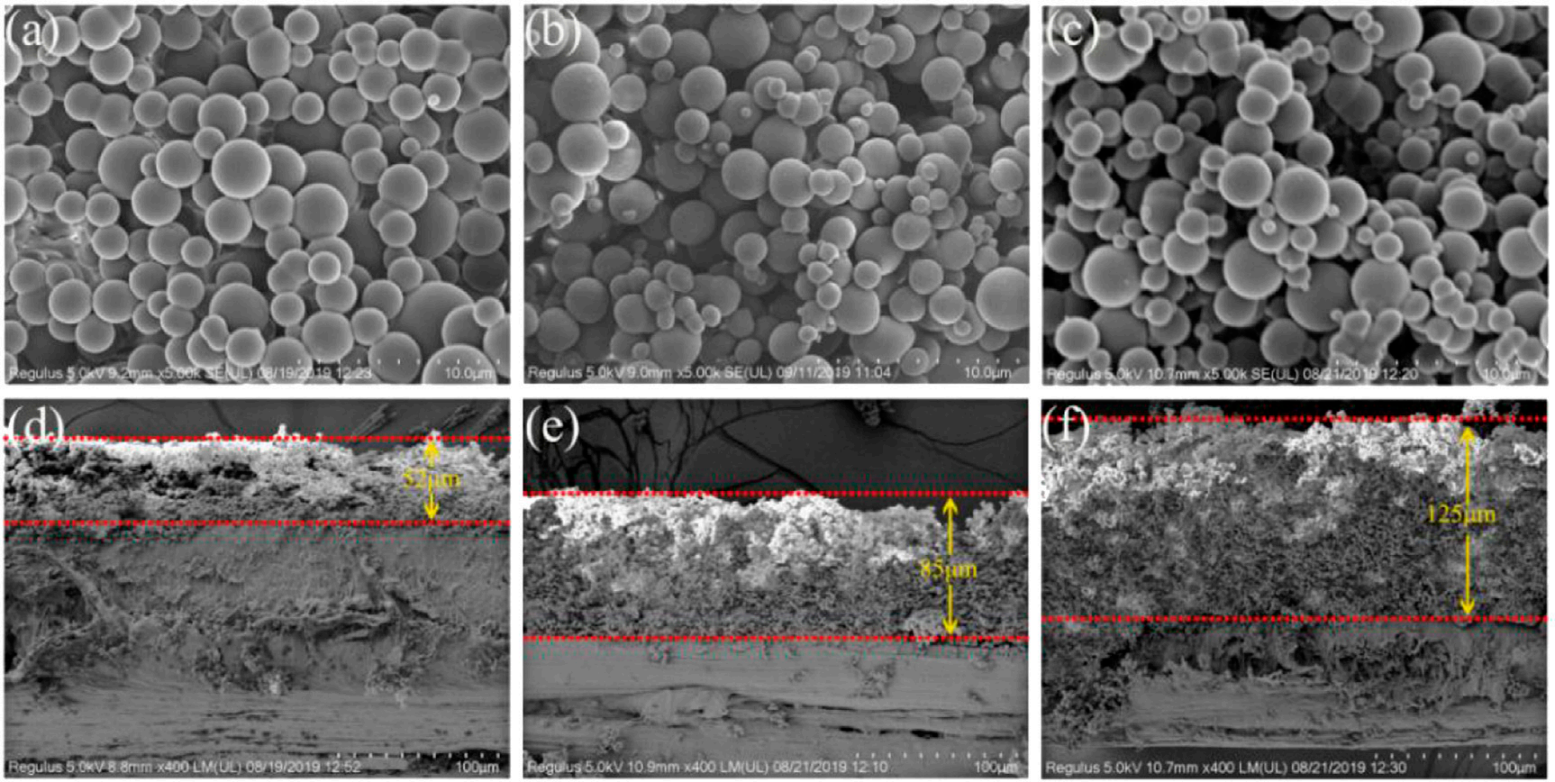
SEM images of h5.0d15-z: **(A)**, **(D)** h5.0d15-10; **(B)**, **(E)** h5.0d15-20; **(C)**, **(F)** h5.0d15-30.

Figures 4A–C shows the SEM images of PVA with different actual degrees of cross-linking. It can be seen that the interconnected PVA microspheres constitute an interconnected porous network structure, the particle size of the PVA microspheres is 1–5 μm. The particle size dispersion of PVA microspheres increases as the actual degree of cross-linking increases. However the overall change is so small that the impact on the pore size is limited. Considering the swelling of the microspheres, it is speculated that the capsule can intercept particles of a few hundred nanometers at least, so it can effectively intercept Cp. It is worth noting that the thickness of the PVA cross-linked porous membrane increases significantly from 52 μm to 125 μm as the content of cross-linking agent increased (Figures 4D–F). The increase in membrane thickness will result in a decrease in membrane flux, which is consistent with the results of membrane flux data in Table 2. Therefore, in addition to the degree of swelling, the membrane thickness is also an important factor affecting the performance of the capsule. The specific surface area, pore size and pore volume of the capsules before and after cross-linked were tested by nitrogen adsorption-desorption experiments, and the results are shown in Supplementary Table S1.

### Performance of cross-linked PVA

The influence of GA content on nominal cross-linking degree and actual cross-linking degree of capsule grid were investigated by Eq. 1 and the intensity of IR absorption peak area, respectively. The results are shown in Table 1. As presented in Table 1, the cross-linker GA content increases from 10 μL to 30 μL, while the corresponding nominal cross-linking degree of PVA increases from 14.71% to 41.26%. This can be due to the increases the aldehyde groups of GA that increases reacted with PVA. However, the nominal cross-linking degree is calculated by weighing methods, and the influence of GA that has not participated in the reaction is not excluded. In the cross-linking reaction system, some functional group does not participate in the cross-linking reaction, so we choose the FTIR peak area of the functional group as a standard. It can be used to verify the degree of cross-linking if some other functional group decreases as the cross-linking degree increases. Lue, et al. (2010) and Farid, et al. (2016) used FTIR method to analyze the actual crosslinking degree of polydimethylsiloxane (PDMS) and PVA cross-linking system, respectively. In this study, actual cross-linking degree of PVA calculated based on FTIR information as shown in Table 1. As can be seen, the actual cross-linking degree is lower than the nominal cross-linking degree, indicating that the prepared cross-linked PVA contains a non-negligible amount of unreacted cross-linking agent.

**TABLE 1:** Nominal cross-linking degree and actual cross-linking degree of h5.0d15-z.

Sample	Nominal cross-linking degree (%	Methylene CH2 2940 cm^−1^	Hydroxyl group OH 3000 and 3600 cm^−1^	Hydroxyl/Methylene ratio	Hydroxyl/Methylene before cross-linking^a^	Hydroxyl reduction (%)	Actual cross-linking degree (%)
h5.0d15-10	20.04	5.89	42.71	7.25	17.55	58.68	14.71
h5.0d15-20	34.66	5.34	35.98	6.74	14.62	53.90	28.60
h5.0d15-30	45.39	4.54	28.49	6.28	12.46	49.65	41.26

^a^ Calculated as (Hydroxyl/methyl)_GA_ × Nominal cross-linking degree + (Hydroxyl/methyl)_PVA_ × (1-Nominal cross-linking degree).

Swelling degree and water flux are other important parameters in cross-linking reaction system, which represent the water uptake capacity and water molecule permeability of the cross-linked product. The data of the swelling degree and the water flux are shown in Table 2. The swelling degree decreases when the cross-linking degree increasing, which is attributed to a decrease in chain length between crosslinks as the content of cross-linker increased. The capsule grid structure became more rigid, and the free volume of the capsule grid decreased (Farid, et al., 2016). Therefore, it has stronger anti-swelling elasticity and reduces the ability of the polymer to absorb water. The expansion of the porous membrane caused the pore wall to shrink and the pores to become narrower, which reduces the permeability (Verhoef, et al., 2008), so the degree of swelling is an important factor affecting membrane flux. Although the degree of swelling decreases with the increase of cross-linker content, the overall difference is no obviously. This is because, on the one hand, the overall degree of cross-linking is relatively high. And on the other hand, the crystallization of the PVA segment will reduce the water absorption in the drying process (the heat treatment process). Theoretically, the water flux will increases with the decrease of the swelling degree. However, the actual measured results are opposite, indicating that the cross-linking agent content has more influence on the membrane performance. That can be further analyzed by SEM.

**TABLE 2:** Swelling degree and Membrane flux of h5.0d15-z.

Sample	h5.0d15-10	h5.0d15-20	h5.0d15-30
Swelling degree	2.99	2.64	2.37
Membrane flux (L·m^−2^·h^−1^)	2750	1530	1150

**FIGURE 5:**
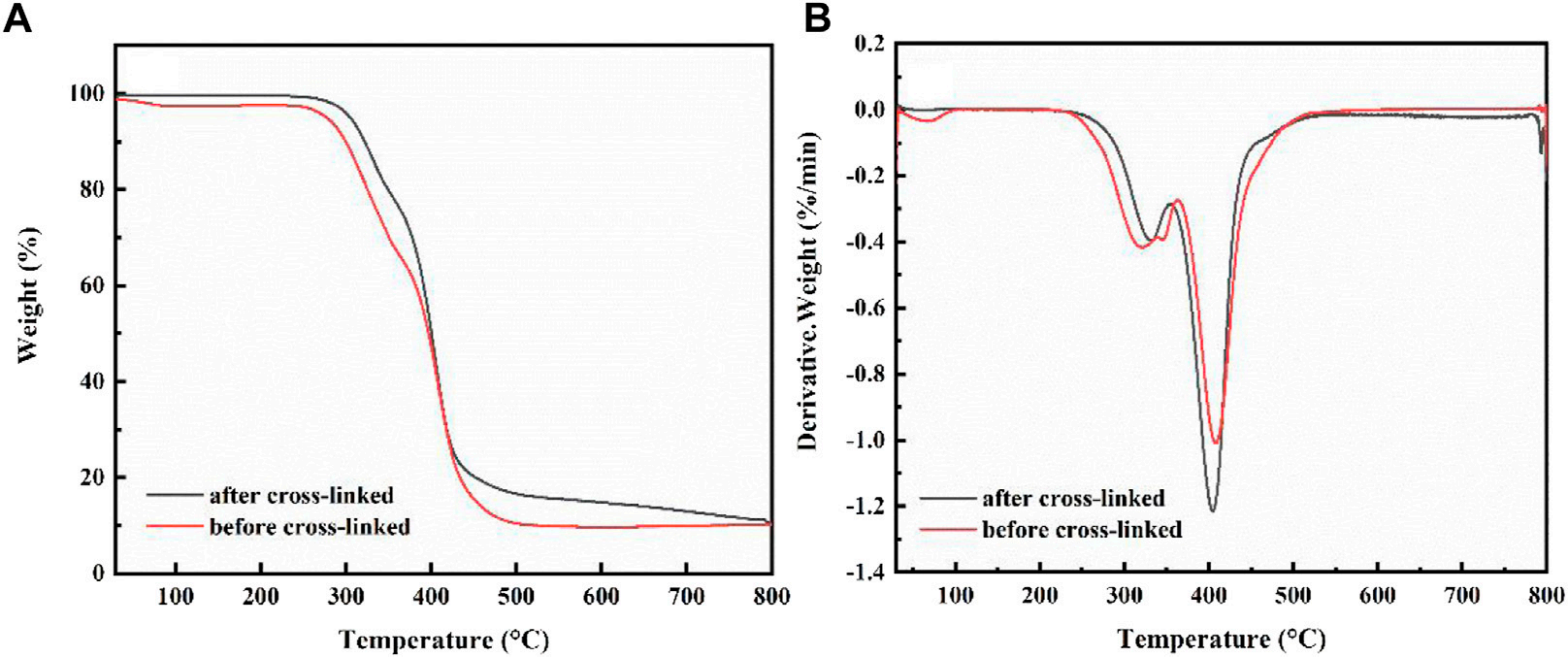
**(A)** TG curves of capsule before and after cross-linked, **(B)** DTG curves of capsule before and after adsorption cross-linked.

Figure 5A displays the TG curves of capsule before and after cross-linked. The same substrate material makes the shapes of the two curves very similar, with only minor differences in temperature. It is evident from TG and DTG curves that PVA capsules decomposition process show two stages: in the first stage, a weak weight loss occurs in the temperature range of 250–350°C, which is probably due to the decomposition of the PVA side chain and hydroxyl breaking dehydration; in the second stage, there is a significant weight loss in the temperature range of 380–450°C, attributing to the breakage of the PVA backbone and decomposition of the base material elastomer in the capsule (Rowe, et al., 2016). Generally speaking, the TG curves of the capsules before and after cross-linking show very similar trends, mainly related to the similarity of the PVA matrix. Notably, compared to the TG curve of the capsule before cross-linking, the decomposition temperature of the capsule of after cross-linked becomes larger and the thermal stability is improved (Liu, et al., 2010).

### Adsorption performance of the cross-linking capsule

**FIGURE 6:**
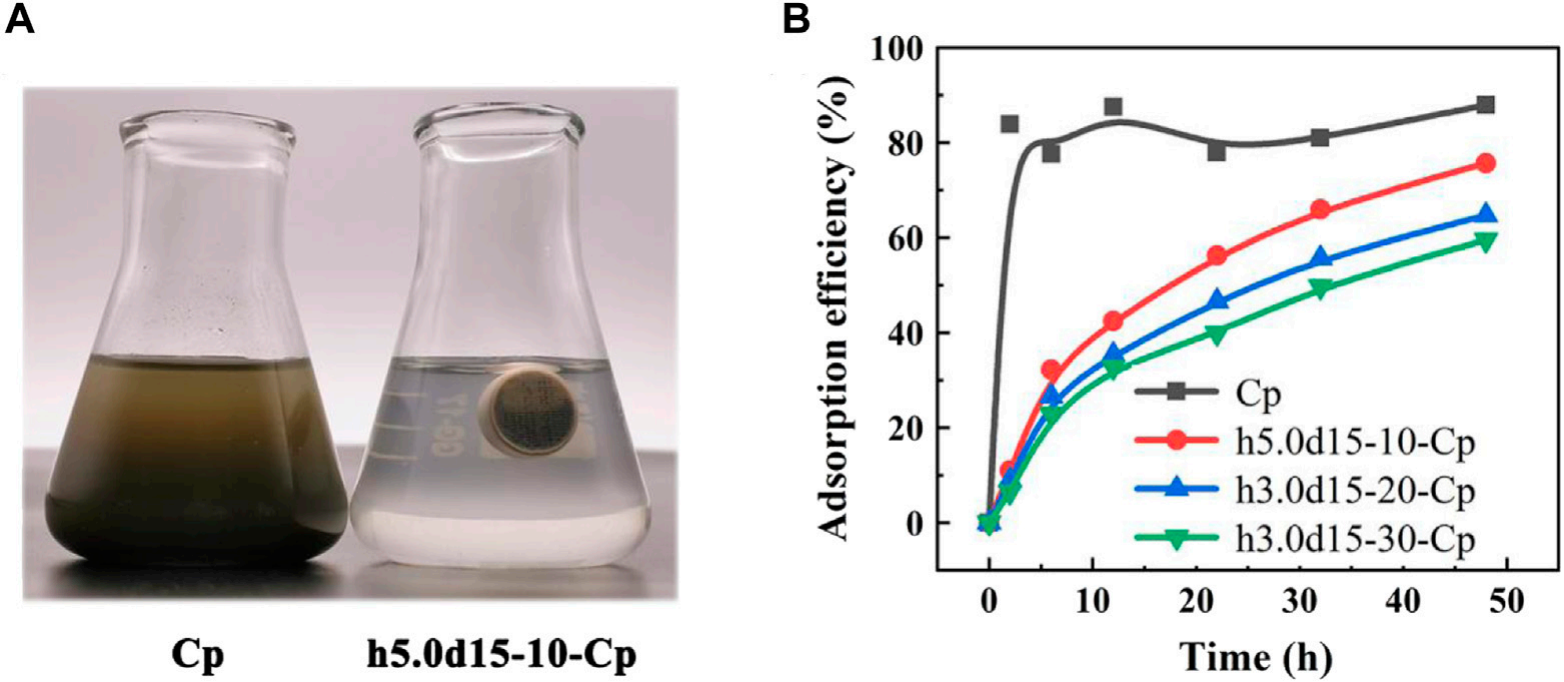
**(A)** Electronic image of Cp powder and h5.0d15-10-Cp samples adsorption experiment; **(B)** Different samples adsorption performance curve.

Previous studies have demonstrated the cross-linking cylindrical capsule allows water molecules to pass through, while blocking Cp. Therefore, the Cp powder, h5.0d15-10-Cp, h5.0d15-20-Cp and h5.0d15-30-Cp were used to remove lead ions in an aqueous solution. It can be seen from Figure 6A that the h5.0d15-10 cylindrical capsule was suspended in the solution, and it was just completely immersed in the solution, effectively avoiding the insufficient contact between the adsorbent and the solution to affect the adsorption performance. However, Cp is dispersed in aqueous solution in powder form and is not conducive reusability and greenly preparation process. The cylindrical capsule adsorbent is self-floating, which is very beneficial for separation and recovery, but self-floating is common in oil-absorbing materials, and there are few reports of adsorbents (An, et al., 2019). In short, 3D printed capsule is a promising adsorption material.

Figure 6B shows the adsorption capability of lead ions on Cp, h5.0d15-10-Cp, h5.0d15-20-Cp and h5.0d15-30-Cp cylindrical capsule. It is obvious that the removal rate of Pb (II) by Cp powder raise rapidly with increasing the adsorption time from initial one to 2 h, the adsorption capability is 83.8%. Then, because of the competitive relationship between adsorption and desorption, the adsorption rate fluctuates slightly with the increase of time. After 48 h, the removal rate is 87.9%. Compared with Cp powder, the adsorption curve of h5.0d15-x-Cp raises slowly with increasing the adsorption time from initial one to 48 h. The maximum adsorption capacity of the h5.0d15-x-Cp cylindrical capsule increases with the cross-linking degree decrease. The maximum adsorption capability of h5.0d15-10-Cp, h5.0d15-20-Cp and h5.0d15-30-Cp cylindrical capsule at 48 h are 75.61%, 64.67% and 59.49% respectively. This was probably because that the permeability of water molecules and lead ions decreases as the thickness of the cross-linking membrane increases, revealing a good removal ability (Kothari, et al., 2022; Shivagangaiah, et al., 2021).

**FIGURE 7:**
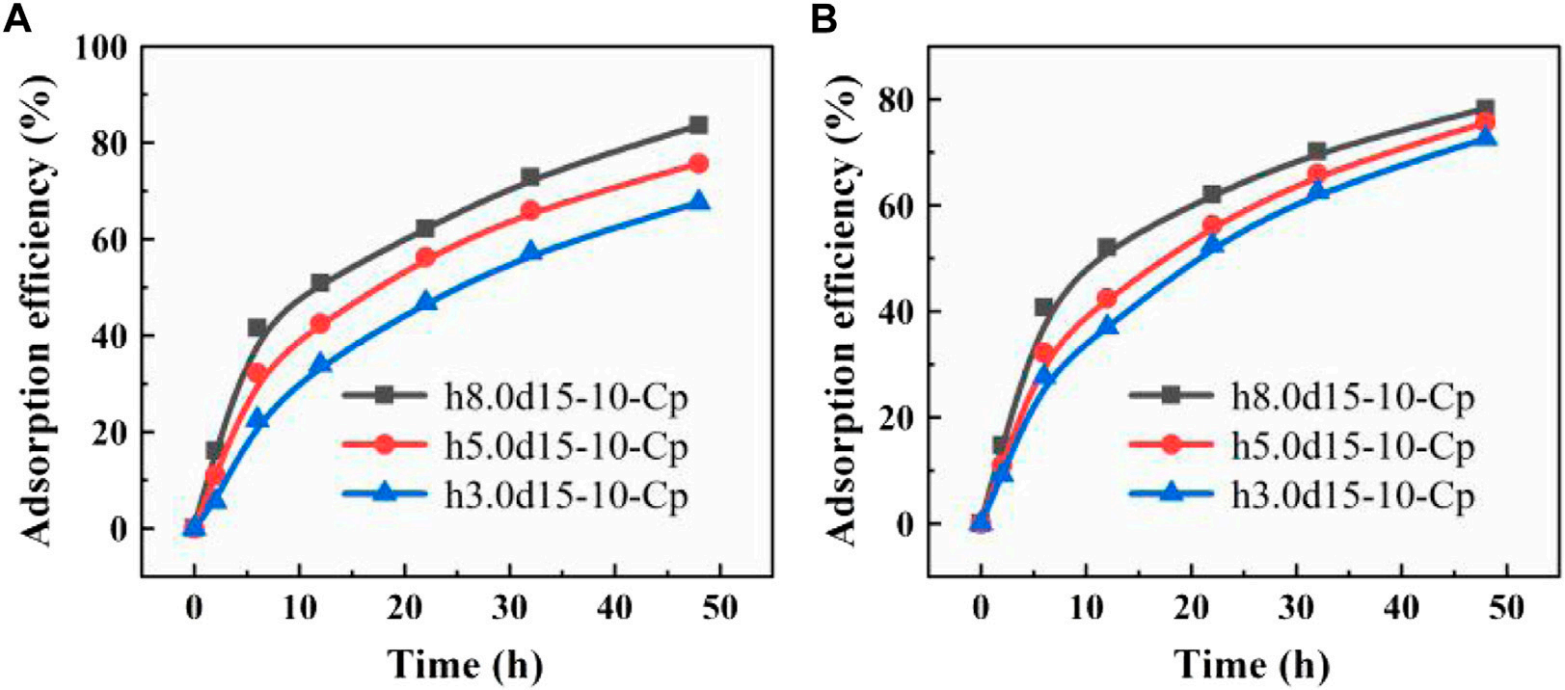
**(A)** Adsorption performance curves of capsules of the same volume and different membrane area; **(B)** Adsorption performance curves of capsules of different volume and same membrane area.

The custom-made is the biggest advantage of the FDM 3D printing. In this study, we designed different cylindrical capsules models: with different diameter (h2.8d20-10-Cp, h5.0d15-10-Cp, h11.3d10-10-Cp), with different height (h3.0d15-10-Cp, h5.0d15-10-Cp, h8.0d15-10-Cp). It can be seen from Figure 7A that since the adsorption of the capsule mainly relies on the PVA porous membrane for internal and external solution exchange, there is no doubt that the adsorption performance of the capsule increases with the increase of the membrane area. The volume of the capsule is also a large influenced factor. As shown in Figure 7B, the larger the volume of the capsule, the better the adsorption performance of the capsule. This may be that the Cp in the smaller volume capsule is not easy to disperse and is easy to agglomerate, which is not conducive to adsorption.

**FIGURE 8:**
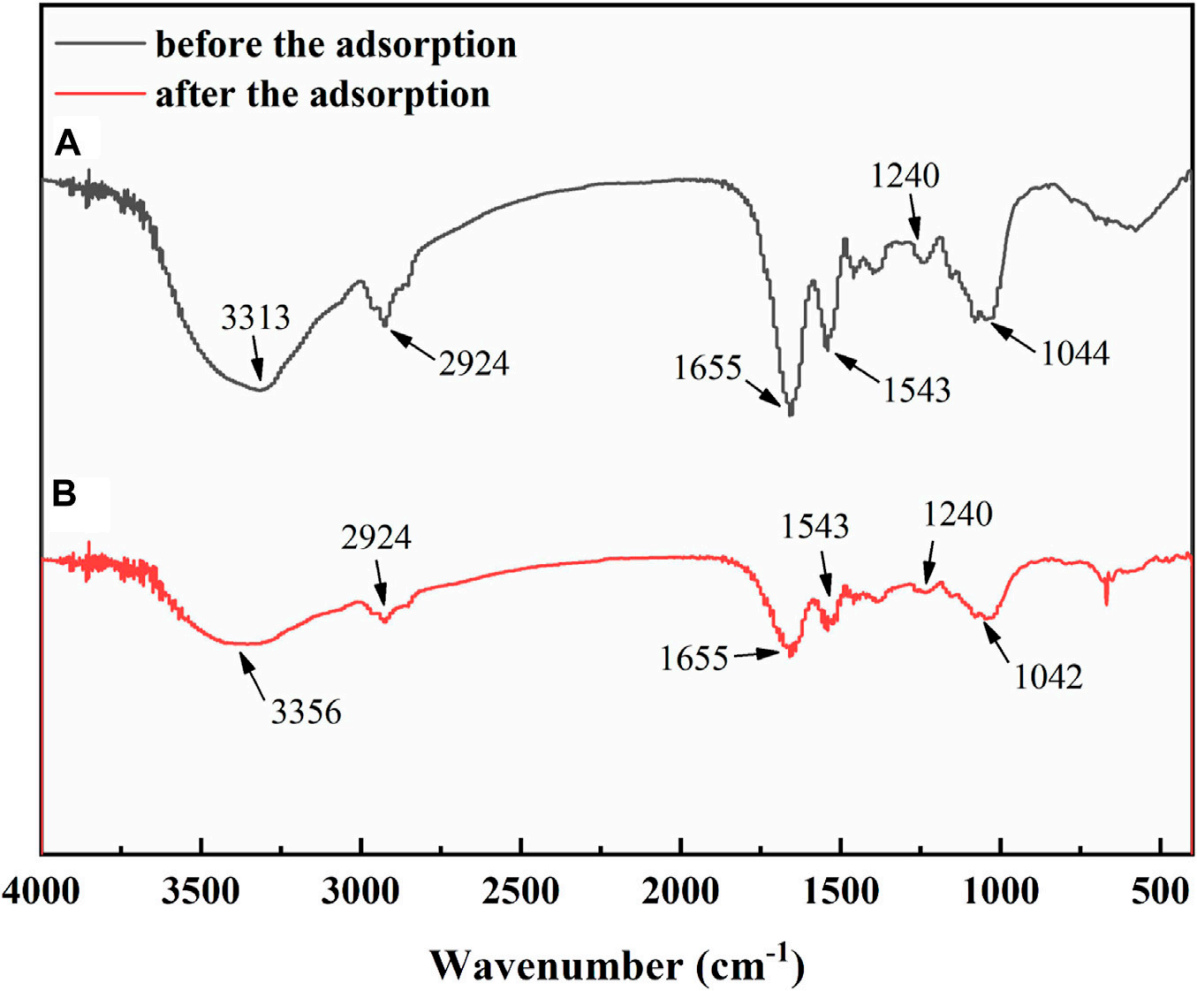
FTIR spectra of CP powder before and after adsorption.

Figure 8 shows the FTIR spectra of CP powder before and after adsorption. It can be seen that a broad and strong absorption peak at 3,313 cm^−1^ are due to O-H stretching vibration of carbohydrates, proteins and lipids in Chlorella. The peak at 2,924 cm^−1^ is considered to be the CH_3_ anti-symmetric stretching vibration and CH_2_ symmetric stretching vibration of lipids and proteins in Chlorella, while the peak at 1,655 cm^−1^ is assigned to the C=O stretching vibration of proteins in Chlorella. The absorption peak at 1,543 cm^−1^ corresponds to N-H deformation vibration of protein in Chlorella and the peak at 1,240 cm^−1^ is the stretching vibration of ester group. At 1,044 cm^−1^ is the absorption peak of P=O stretching vibration of phospholipids, DNA and RNA in Chlorella. Comparing the curves of Chlorella before and after Pb^2+^ adsorption, the O-H stretching vibration absorption peak at 3,313 cm^−1^ shifted to 3,356 cm^−1^, which shows a blue shift due to the some of the hydroxyl groups participated in the adsorption and were occupied by Pb^2+^, resulting in the partial breakage of the formed hydrogen bonds. This shift indicates that the hydroxyl group has a remarkable contribution in the adsorption of Pb^2+^ by Chlorella. In addition, the SEM images in Supplementary Figure S2 also shows that the surface of CP powder relatively rough with some pits, which is conducive to the combination with lead ions in solution and enhance the adsorption efficiency.

### Recycling performance

**FIGURE 9:**
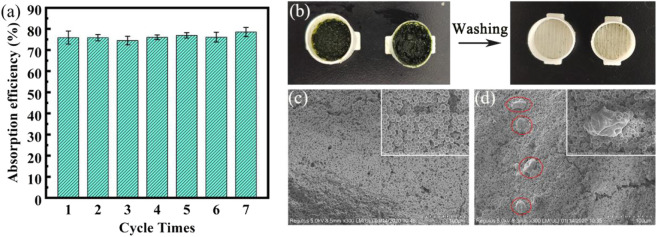
**(A)** Cyclic adsorption performance chart; **(B)** Capsule diagram after adsorption; **(C,D)** SEM images of capsules before and after adsorption.

In order to test the recycling performance of the cylindrical capsule, we added two “ears” to the cylindrical capsule for easy unpacking (Supplementary Figure S3). The repeated experiment is to take out the adsorbed Cp from the capsule, rinse the capsule shell, and then re-encapsulate the fresh Cp powder in the capsule. As shown in Figure 9A, the performance of the capsule is basically unchanged after 7 cycles of use, and the 48 h removal rate can still reach 78.56%. We can also see from Figures 9C,D that the micro-morphology of the capsules has not changed significantly before and after use. The PVA hydrogel membranes are obviously detached or damaged due to repeated use, but there is only a small amount of Cp remaining on the surface without being washed out.

## Conclusion

In conclusion, we designed cylindrical capsules to encapsulate adsorbent powder by 3D printing technology with cross-linked PVA hydrogels membrane. These 3D printing cylindrical capsules possessing the self-floating performance were prepared for removal of lead ions. The results showed that as the amount of cross-linking agent increased, the thickness of the PVA hydrogels membrane increased, thereby reducing the water flux. Adsorption experiments showed that larger capsule volume and membrane area are more favorable for adsorption. After seven cycles of use, the capsules retain their original properties, and the 48 h removal rate can still reach 78.56%. Our work proves that the application of self-floating 3D printed capsules as auxiliary tools in wastewater treatment, chemical slow release, sample preparation and separation, and other fields has broad application prospects.

## Data Availability

The original contributions presented in the study are included in the article/Supplementary Material, further inquiries can be directed to the corresponding authors.

## Supplementary material

SUPPLEMENTARY FIGURE S1 (A,B) SEM images of capsule before cross-linked, (C,D) SEM images of capsule after cross-linked.

SUPPLEMENTARY FIGURE S2 (A,B) SEM images of CP powder before adsorption of lead ions, (C,D) SEM images of CP powder after adsorption of lead ions.

SUPPLEMENTARY TABLE S1 Results of BET experiment.
