# Supplementary material for: 3D printed cylindrical capsules as a Chlorella pyrenoidosa immobilization device for removal of lead ions contamination
Source: Front Chem. 2022 Nov 30;10:987619. doi: 10.3389/fchem.2022.987619 (PMC9748691; doi:10.3389/fchem.2022.987619)
Supplement: Supplementary file 1 [file DataSheet1.pdf]

Supporting Information

3D printed cylindrical capsules as a Chlorella pyrenoidosa Immobilization device for removal of lead ions contamination

Shuzhen Lan[1,2], Xinshu Xia[1,3*], Zhen Liu[1,2], Yujin Yang[1,3], Qingrong Qian[1,3], Yongjin Luo[1,3], Qinghua Chen[1,3], Changlin Cao[1,3*], Liren Xiao[1,2*]

[1]Engineering Research Center of Polymer Green Recycling of Ministry of Education, Fujian Normal University, Fuzhou 350007, China

[2]College of Chemistry and Materials, Fujian Normal University, Fuzhou 350007, China

[3]College of Environment and Resources, Fujian Normal University, Fuzhou 350007, China

*** Correspondence:**
Xinshu Xia, Changlin Cao, Liren Xiao
xsxia@fjnu.edu.cn, caochlin3@fjnu.edu.cn, xlr1966@fjnu.edu.cn

Figure S1 shows the microstructure of the PVA capsules before and after cross-linking. It can be seen from the figure that the surface of the capsule before cross-linking is flat and smooth, and no excess round particles appears. While the surface of the cross-linked capsule is filled with PVA microspheres, a large number of PVA microspheres are connected to each other and arranged closely to cover the matrix. Most of the PVA microspheres have a particle size below 5 μm, and the tight junctions between the microspheres form a porous network of interconnected channels. These characteristics are considered to be beneficial for free exchange of adsorbent between internal and external solutions through the protective film formed by the microspheres.

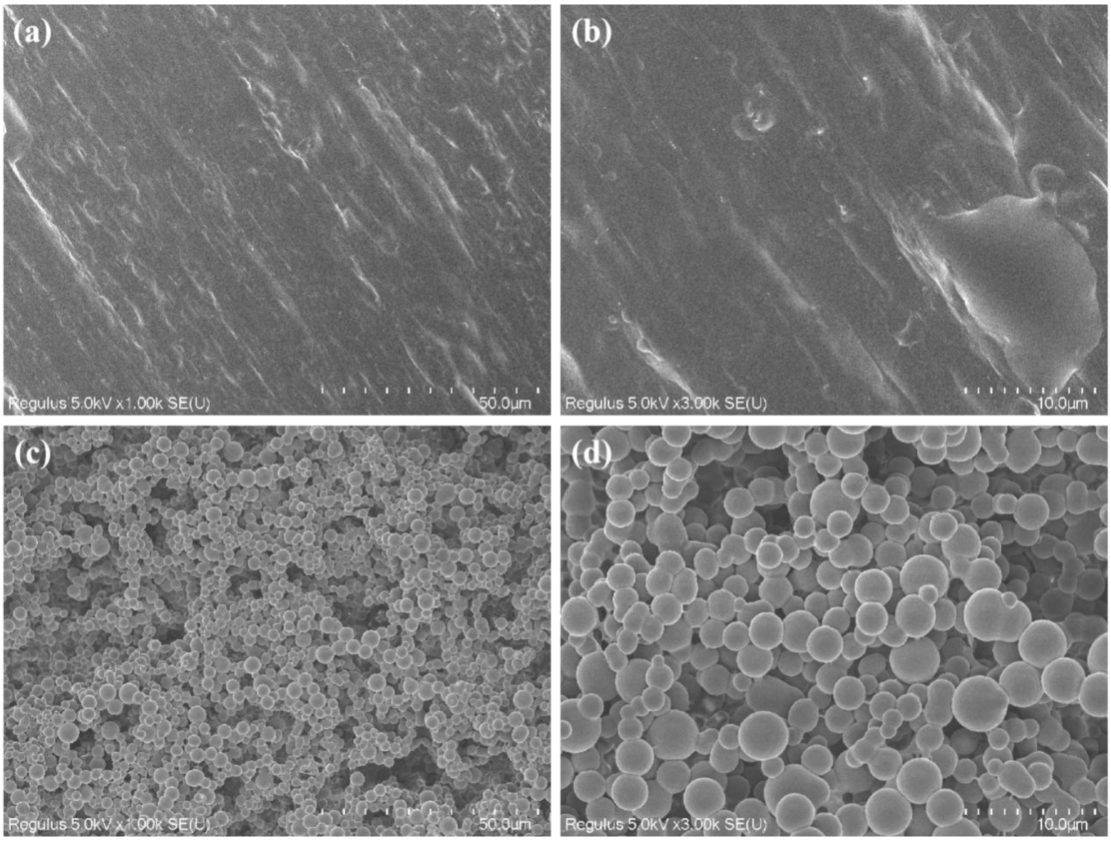

Figure S1. (a-b) SEM images of capsule before cross-linked, (c-d) SEM images of capsule after cross-linked

The specific surface area, pore size and pore volume of the capsules before and after cross-linked were tested by nitrogen adsorption-desorption experiments, and the results are shown in Table S1. It can be seen that the specific surface area of the capsule is small and the difference before and after cross-linked is not significant. The BJH model was used to calculate the pore size distribution, and it was concluded that the pore size distribution of the capsules after cross-linked was concentrated around 15 nm, which was about 5 times than the pore size before cross-linked, and the pore volume was enlarged by about 4 times. This result indicates that cross-linked increases the pore size and pore volume of the capsule to some extent, and the specific surface area is slightly increased. These characteristics facilitate the free exchange of the adsorbent between internal and

external solutions and also prevent the leakage of the powder-based adsorbent.

Table S1 Results of BET experiment

sample	$a_{s,\ BET}$ /m^2. g $^{-1}$	Total pore volume(p/p$_0$=0.0995)/cm^3. g^{-1}	Mean pore diameter/nm
Before cross-linked	1.1220	0.001060	3.7776
After cross-linked	1.4441	0.005557	15.3918

Figure S2 shows the SEM images of CP powder before and after the adsorption of lead ions. It can be seen from the Figure S2a and b that CP powder is roughly in the shape of a round sphere with different sizes, and the surface of CP powder relatively rough with some pits, and this surface property is conducive to the combination with lead ions in solution and enhance the adsorption efficiency. As shown in the Figure S2 (c-d), the CP powder after adsorption of lead ions occurs agglomeration resulting in larger particle size and more irregular morphology, which may be due to the swelling caused by some adsorption of lead ions by CP powder.

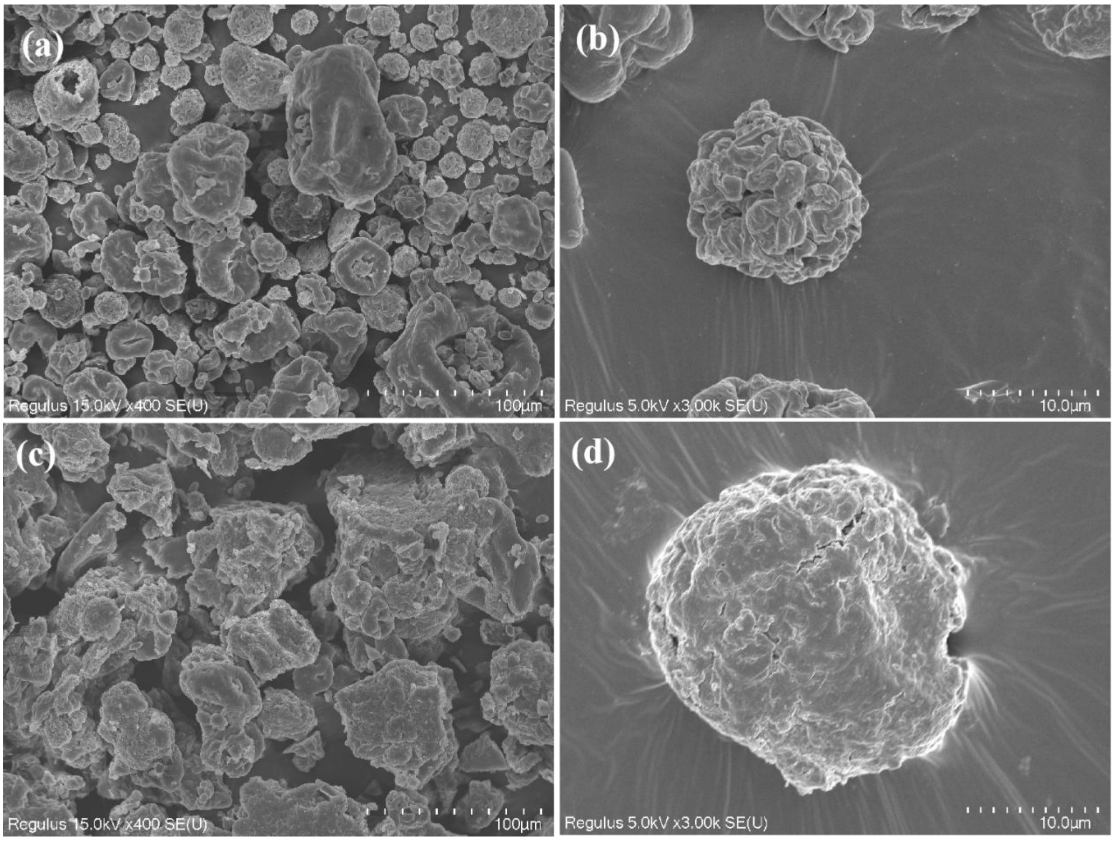

Figure S2. (a-b) SEM images of CP powder before adsorption of lead ions, (c-d) SEM images of CP powder after adsorption of lead ions

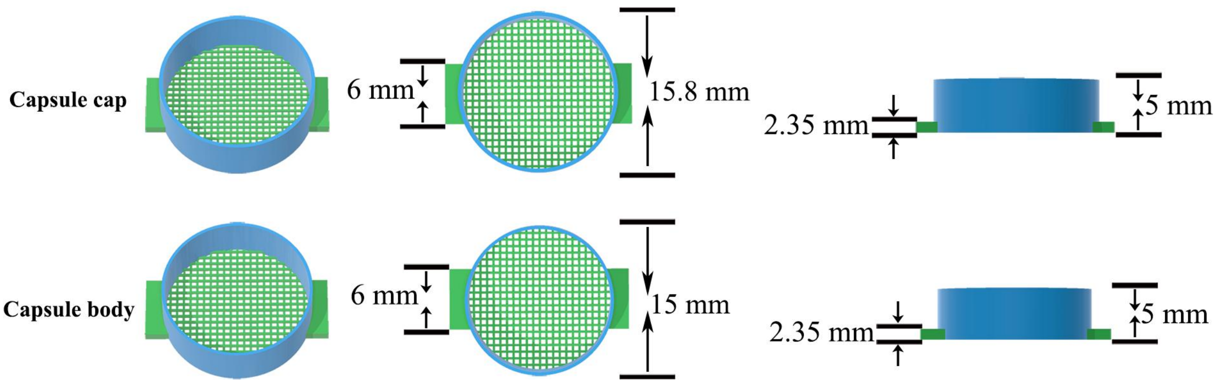

Figure S3. Schematic illustration of the cylindrical capsule models with "ears" structures
